# NRICM101 ameliorates SARS-CoV-2–S1-induced pulmonary injury in K18-hACE2 mice model

**DOI:** 10.3389/fphar.2023.1125414

**Published:** 2023-06-21

**Authors:** Wen-Chi Wei, Keng-Chang Tsai, Chia-Ching Liaw, Chun-Tang Chiou, Yu-Hwei Tseng, Geng-You Liao, Yu-Chi Lin, Wen-Fei Chiou, Kuo-Tong Liou, I-Shing Yu, Yuh-Chiang Shen, Yi-Chang Su

**Affiliations:** ^1^ National Research Institute of Chinese Medicine, Ministry of Health and Welfare, Taipei, Taiwan; ^2^ Ph.D Program in Medical Biotechnology, College of Medical Science and Technology, Taipei Medical University, Taipei, Taiwan; ^3^ Department of Biochemical Science and Technology, National Chiayi University, Chiayi, Taiwan; ^4^ Institute of Physiology, School of Medicine, National Yang Ming Chiao Tung University, Taipei, Taiwan; ^5^ Laboratory Animal Center, National Taiwan University College of Medicine, Taipei, Taiwan

**Keywords:** COVID-19, NRICM101, traditional Chinese medicine, lung injury, pattern recognition receptors

## Abstract

The coronavirus disease 2019 (COVID-19) pandemic continues to represent a challenge for public health globally since transmission of different variants of the virus does not seem to be effectively affected by the current treatments and vaccines. During COVID-19 the outbreak in Taiwan, the patients with mild symptoms were improved after the treatment with NRICM101, a traditional Chinese medicine formula developed by our institute. Here, we investigated the effect and mechanism of action of NRICM101 on improval of COVID-19-induced pulmonary injury using S1 subunit of the SARS-CoV-2 spike protein-induced diffuse alveolar damage (DAD) of hACE2 transgenic mice. The S1 protein induced significant pulmonary injury with the hallmarks of DAD (strong exudation, interstitial and intra-alveolar edema, hyaline membranes, abnormal pneumocyte apoptosis, strong leukocyte infiltration, and cytokine production). NRICM101 effectively reduced all of these hallmarks. We then used next-generation sequencing assays to identify 193 genes that were differentially expressed in the S1+NRICM101 group. Of these, three (*Ddit4*, *Ikbke*, *Tnfaip3*) were significantly represented in the top 30 enriched downregulated gene ontology (GO) terms in the S1+NRICM101 group versus the S1+saline group. These terms included the innate immune response, pattern recognition receptor (PRR), and Toll-like receptor signaling pathways. We found that NRICM101 disrupted the interaction of the spike protein of various SARS-CoV-2 variants with the human ACE2 receptor. It also suppressed the expression of cytokines IL-1β, IL-6, TNF-α, MIP-1β, IP-10, and MIP-1α in alveolar macrophages activated by lipopolysaccharide. We conclude that NRICM101 effectively protects against SARS-CoV-2-S1-induced pulmonary injury via modulation of the innate immune response, pattern recognition receptor, and Toll-like receptor signaling pathways to ameliorate DAD.

## Graphical Abstract

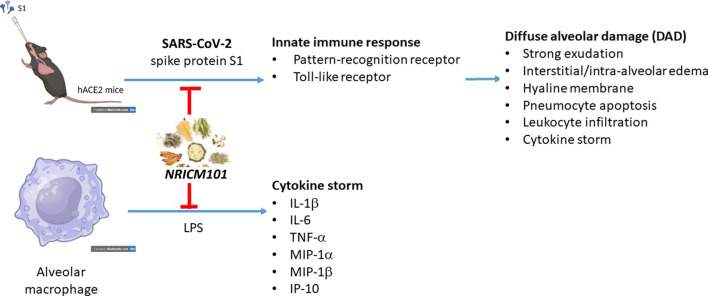

## 1 Introduction

The coronavirus disease 2019 (COVID-19) pandemic has been recognized as a global health emergency, and reinfections with COVID-19 continue to be reported globally, which may be partially due to mutations of the virus (Harvey et al., 2021; Indari et al., 2021; Ren et al., 2022). COVID-19 is caused by infection with severe acute respiratory syndrome coronavirus 2 (SARS-CoV-2) (Lu et al., 2020; Zhou et al., 2020; Zhu et al., 2020). The virus is transmitted mainly via inhaled droplets and aerosols or via direct exposure to mucus or body fluid from infected symptomatic cases (Amirian, 2020).

Although it can affect a wide range of organs, the respiratory system is the primary target of SARS-CoV-2 infection (Guan et al., 2020; Huang et al., 2020; Wang et al., 2020; Xu et al., 2020; Zhu et al., 2020), and SARS-CoV-2-induced acute respiratory distress syndrome (ARDS) has been suggested to be a major threat to the patient’s life. Infection with SARS-CoV-2 causes abnormal lung tissue inflammation, pulmonary oedema, and immune cell infiltration into the lung tissues, leading to lung damage, shortness of breath, and ARDS (Grasselli et al., 2020; Sinha et al., 2020; Tay et al., 2020; Reddy et al., 2021). Angiotensin-converting enzyme 2 (ACE2), which is highly expressed in pulmonary epithelial cells (Li et al., 2003; Gheblawi et al., 2020), has been identified as responsible for the attachment of SARS-CoV-2 to the host (Zhou et al., 2020; Beyerstedt et al., 2021). The virus infects the alveolar type 2 epithelial cells (AT2 cells) via ACE2, adjacent alveolar epithelial cells are then infected in the same manner by newly released viral particles, and this leads to the loss of both AT1 and AT2 cells, causing alveolar damage and eventually ARDS (Mulay et al., 2021; Lamers and Haagmans, 2022). Pathological findings show that inflammation-associated diffuse alveolar damage (DAD) may underlie COVID-19-associated pneumonia and ARDS. Further, SARS-CoV-2-infected cells secret cytokines such as interleukins (e.g., IL-1 and IL-6), tumor necrosis factor-α (TNF-α), interferons (IFNs), chemokine ligands (CXCLs), and monocyte chemoattractant proteins (MCPs) (Smail et al., 2021; Lamers and Haagmans, 2022). The immune system overreaction triggered by SARS-CoV-2 causes acute and excessive production of proinflammatory cytokines, especially in the lung tissue, the so-called “cytokine storm”, which may contribute to the lethality of COVID-19 (Ragab et al., 2020; Montazersaheb et al., 2022). Among the types of immune cells found in the alveoli after infection, macrophages have been reported to play a critical role in SARS-CoV-2-associated ARDS and the cytokine storm (Rendeiro et al., 2021; Zhang, et al., 2021). The purpose of the virus-induced inflammatory response is to defend against the invading virus particles; however, the overreaction of the immune system instead causes the subsequent inflammation and lung injury. Suppression of lung inflammation and injury is therefore an effective supportive therapeutic approach for COVID-19 patients.

Currently, several traditional Chinese medicine (TCM)-based formulations are used to treat COVID-19 in Taiwan, including NRICM101 (Tsai et al., 2021), Jing Si Herbal Tea (Hsieh et al., 2022), and Jing Guan Fang (Ping et al., 2022). Under an emergency-use authorization, NRICM101 is a prescription TCM formula commonly used in Taiwan to treat COVID-19 and the associated pulmonary disorders. Our previous study showed that it significantly reduced the formation of plaques that is associated with SARS-CoV-2, disrupted the interaction between the SARS-CoV-2 spike protein and the ACE2 receptor, and inhibited coronavirus 3C-like protease activity (Tsai et al., 2021). In addition, it exhibited a significant regulatory effect on the lipopolysaccharide (LPS)-stimulated secretion of the critical proinflammatory cytokines IL-6 and TNF-α in murine alveolar macrophages.

Clinical observations show that use of NRICM101 alleviates fever, enhances cardiopulmonary function, and reduces the risk of developing severe disease (Tseng et al., 2022). Moreover, chest X-rays showed that NRICM101 administration caused the focal ground-glass opacities to dissipate in most COVID-19 patients. Since NRICM101 possesses important properties for curbing COVID-19 progression, it may also be able to ameliorate lung inflammation and thus improve ARDS.

## 2 Materials and methods

### 2.1 Pulmonary injury in K18-hACE2 mice caused by administering SARS-CoV-2 spike protein S1

There were three treatment groups: sham (sham surgery and saline), S1+saline, and S1+NRICM101. We administered SARS-CoV-2 S1 intratracheally to the mice as described elsewhere (Wei, et al., 2022). Briefly, we anesthetized each mouse by injecting it with xylazine (6 mg/kg) and ketamine (60 mg/kg) intraperitoneally. We then made a small incision in the skin of its neck. We dissolved the S1 (400 μg/kg in 2 mL/kg) in normal sterile saline solution and administered it gradually into the animal’s tracheal lumen via the incision. We then closed the incision and the animal was given time for recovery. Each day for 3 days after this procedure, we administered either NRICM101 (3.0 g/kg) or saline solution (the control) to orally the mice. Three days later, we euthanized the mice and measured SO_2_ (the concentration of oxygen relative to its maximum possible concentration) in the lungs using an iSTAT G3+ detection kit (Abbott Point of Care, Mississauga, ON, Canada). Finally, we analyzed these results using a video-tracking system (SMART v2.5.21, Panlab, Cornellà, Spain) and calculated the immediate (day 0 after S1 administration) and 72 h (day 3 after S1 administration) rates of survival.

### 2.2 Histopathology and immunohistochemistry

Fifteen to twenty ∼30 μm serial sections were collected from the same part of the lung of mice from all treatment groups for immunohistochemical analysis. Before staining with specific antibodies, we prepared the tissue sections using the general protocol featuring fixation, permeabilization, and blocking. We then randomly selected tissue slices and incubated them overnight at 4°C in phosphate-buffered saline (PBS) containing 3% albumin and stained them for specific protein markers using primary antibodies as follows: SARS-CoV-2 spike protein subunit 1 (S1) RBD (1:100), AT1 (PDPN, 1:100), AT2 (SFTPC, 1:100), F4/80 (macrophage; 1:100), Ly6G (1:100), MPO (1:100), IL1β (1:100), IL-6 (1:100), CD8 (1:100), and TLR4 (1:100) (all from GeneTex, Irvine, CA, United States); CD11b (1:50; Abcam, Cambridge, United Kingdom); the active form of caspase 3 (cCasp3, 1:50; Santa Cruz Biotechnology, Dallas, TX, United States); and phospho(p)-P65NFκB (1:50; BD, San Diego, CA, United States). We washed the tissue sections thoroughly and then stained them with secondary antibodies conjugated with Alexa Fluor 488, 555, or 647 (Cell Signaling Technology, Danvers, MA, United States). We mounted the correctly stained sections on coverslips in medium containing 4′,6-diamidino-2-phenylindole (DAPI) and imaged them using a confocal Zeiss LSM780 laser-scanning microscope (Carl Zeiss, Jena, Germany). Using Zen 2011 (black edition, Carl Zeiss MicroImaging, 1997–2011) and AlphaEase FC (Alpha Innotech, San Leandro, CA, United States) we identified, counted, and calculated the area covered by the immunopositive cells or identified the immunopositive areas and estimated the proportion of the total area they comprised (as a percentage). We applied this procedure to the whole field of view in regions of interest that we sampled from each group. We used 30–1×00 magnification and performed 3–5 independent replicates of each experiment. Finally, we used Masson’s trichrome staining method to detect tissue fibrosis.

### 2.3 RNA sequencing and RNA-seq data analysis

We checked the quality and amount of the RNA samples using a SimpliNano Spectrophotometer (Biochrom, Holliston, MA, United States) and assessed the degradation and integrity of the RNA using a Qsep 100 DNA/RNA Analyzer (BiOptic, New Taipei City, Taiwan). We created sequence libraries using the total RNA with a KAPA mRNA HyperPrep Kit (KAPA Biosystems, Roche, Basel, Switzerland); we produced the raw data via high-throughput sequencing using the NovaSeq 6000 platform (Illumina, San Diego, CA, United States) and ascertained its quality with the FastQC and MultiQC software. We used the Trimmomatic v0.38 tool to produce high-quality raw paired-end read data, which we used in all later analyses. We used HISAT2 v2.1.0 to align the read pairs to the reference genome and then FeatureCounts v2.0.0 to count the number of reads that had been mapped to individual genes. We then used DEGseq v1.40.0 and DESeq2 v1.26.0 to identify DEGs and clusterProfiler v3.14.3 to conduct the GO functional annotation and assess the KEGG pathway enrichment. Finally, we built a protein–protein interaction (PPI) network of the DEGs with STRINGdb (https://string-db.org/).

### 2.4 BLI (bio-layer interferometry) assay

We conducted a BLI assay according to the previously published procedure (Wei et al., 2022). Briefly, we immobilized several recombinant SARS-CoV-2 variant RBD proteins (GeneTex) on HIS1K sensor tips for 300 s at 50 μg/mL in PBS. We then conducted sequential sample testing, performing the baseline, association, and dissociation steps at 60 s, 180 s, and 180 s, respectively. We used the association signal to align the data and fitted the curves using a 1:1 best-fit model using FortéBio’s data analysis software (Sartorius, FortéBio^®^).

### 2.5 ACE2-spike protein inhibition ELISA

We conducted an ELISA according to the previously published procedure (Wei et al., 2022). Briefly, we coated the recombinant SARS-CoV-2 variant RBD proteins (0.1–2 μg/well, GeneTex) onto a microplate. We added serial dilutions of NRICM101 (1/10×, 1/50×, 1/100×, 1/150×, 1/300×, 1/600×, 1/900×, 1/1200×, 1/1500×, 1/2000×, 1/3000×, and 1/6000×) to the wells and incubated the microplate at 37°C. We then added recombinant human ACE2 protein (0.2 μg/mL; Sino Biological, Beijing, China) to the wells and incubated the plate for 40 min at 37°C. Finally, we incubated it with rabbit anti-human IgG-HRP for a further 40 min, and then quantified the intensity of the signal as optical density at 450 nm using a SPECTROstar Nano microplate reader (BMG LABTECH, Ortenberg, Germany).

### 2.6 Cytokine inhibition assay

For this assay, we cultured a total of 5 × 10^5^ MH-S CRL-2019 murine alveolar macrophages (ATCC, Manassas, VA, United States) in 12-well culture plates for 24 h. We then treated the cells with various dilutions of NRICM101 in the presence of LPS (1 μg/mL). We prepared the supernatant for cytokine identification and used a Proteome Profiler Mouse Cytokine Array Kit (R&D Systems, Minneapolis, MN, United States), which can detect 40 types of cytokine, to detect cytokine expression.

## 3 Results

### 3.1 NRICM101 ameliorated SARS-CoV-2 spike protein S1-induced pulmonary injury and reduction in lung oxygen saturation (SO_2_) in K18-hACE2 mice

**FIGURE 1:**
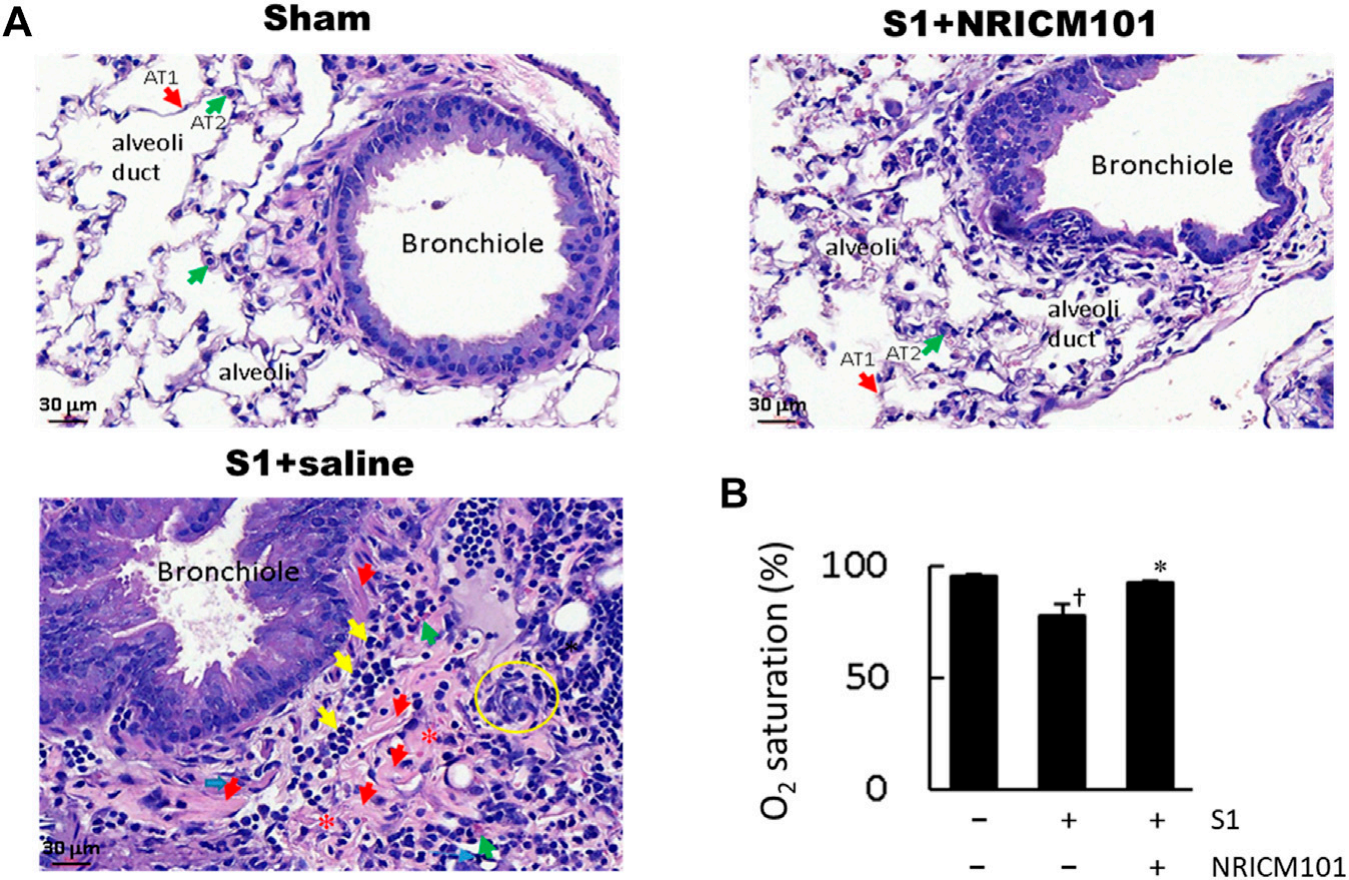
Effects of NRICM101 on lung injury symptoms induced by administering SARS-CoV-2 spike S1 protein to K18-hACE2 mice. **(A)**, Hematoxylin and eosin (H&E) staining of lung tissue from the three treatment groups. In the S1+saline group, the hallmarks of diffuse alveolar damage (DAD) can be observed: hyaline membranes (red arrows), pneumocyte hyperplasia (yellow circle), interstitial and alveolar edema (loss of alveoli, red stars), inflammatory cell infiltrate (yellow arrows). These were not observed in the sham (control) or S1+NRICM101 groups. **(B)**, Quantitative summary of the arterial blood oxygen saturation (SO_2_) in the three groups. Data presented are the mean ± standard error of the mean (SEM) (n = 5 per group). ^†,^* *p* < 0.05 compared with the sham or S1+saline group, respectively; one-way analysis of variance (ANOVA) followed by Student–Newman–Keuls (SNK) *t*-test. We conducted these experiments using hACE2 transgenic mice. The S1 protein was administered intratracheally (400 μg/kg) and the NRICM101 was administered orally (3.0 g/kg). We divided the mice into three treatment groups: sham surgery and saline control (sham), S1 protein administration and vehicle (saline) treatment (S1+saline), and S1 protein administration and NRICM101 treatment (S1+NRICM101).

We investigated the effect of NRICM101 on protection against SARS-CoV-2-associated pulmonary injury using SARS-CoV-2 spike protein S1-induced lung injury in the K18-hACE2 mouse model (Colunga Biancatelli et al., 2021). The mice were pretreated with the S1 for 2 h and then treated with NRICM101 (3.0 g/kg). Hematoxylin and eosin (H&E) staining and microscopic analysis indicated that the S1 had induced acute DAD, as characterized by hyaline membranes lining the alveolar spaces, hyperplastic pneumocytes, the loss of alveolar epithelial cells into the adjacent spaces, interstitial and alveolar edema, congestion and hemorrhaging in the alveolar septa (which contained cellular debris and infiltrating mononuclear inflammatory cells), none of which were evident in the sham group (Figure 1A). Treatment with NRICM101 markedly reduced this S1-induced acute lung injury, as evidenced by reductions in the DAD markers. We also examined the effect of NRICM101 on lung oxygen saturation (SO_2_), which is associated with pulmonary function. As suggested by our histological observations, NRICM101 significantly restored the S1-induced decrease in lung SO_2_ (Figure 1B; from 78.0% ± 4.6% to 92.6% ± 0.5%). Together, these results suggest that NRICM101 effectively attenuated the S1-induced pulmonary injury, thereby improving lung SO_2_, in hACE2 mice.

### 3.2 NRICM101 reduced SARS-CoV-2 spike protein S1-induced alveolar cell apoptosis in hACE2 mice

**FIGURE 2:**
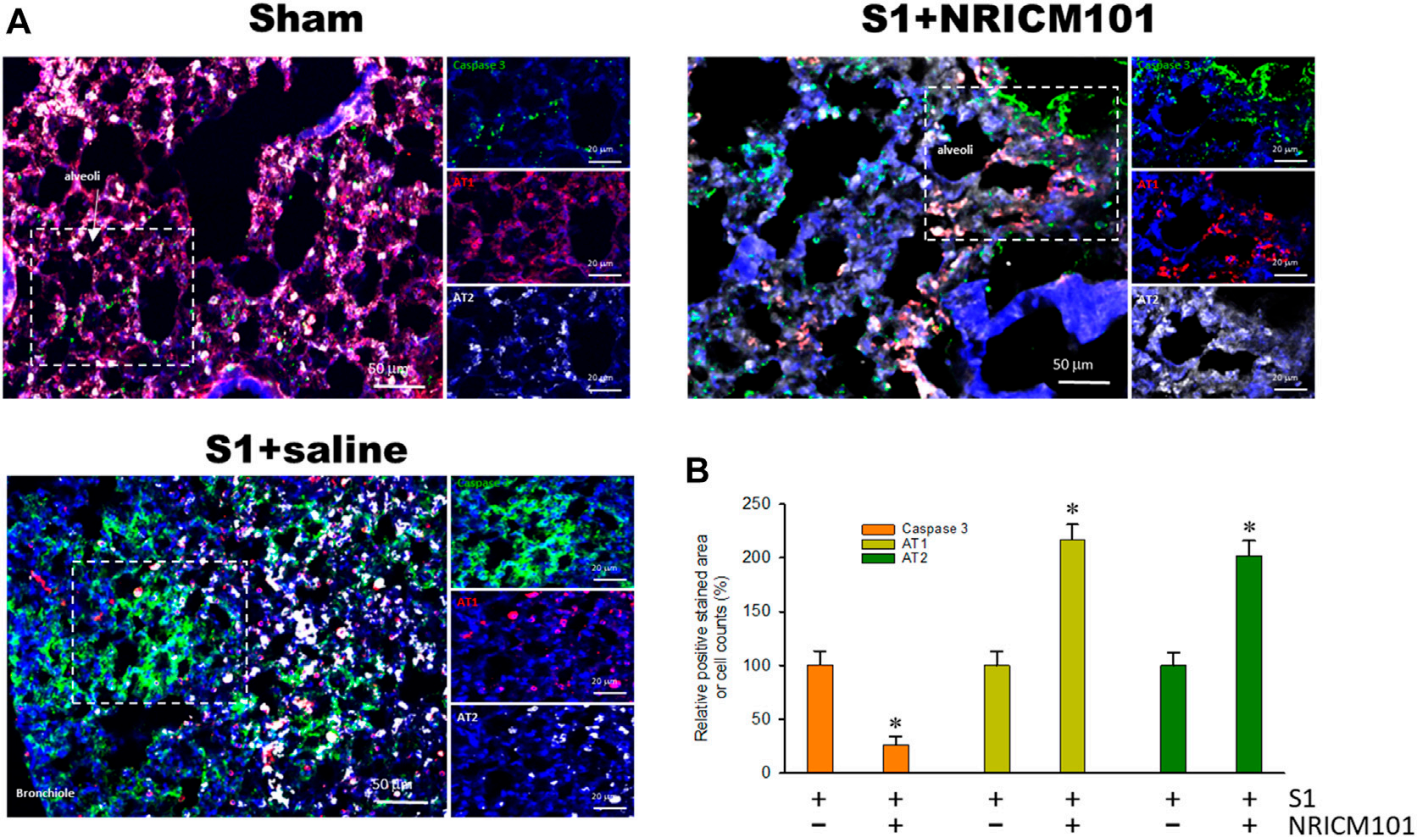
The effects of NRICM101 after 72 h on S1-induced alveolar cell apoptosis in K18-hACE2 mice with lung injury symptoms induced by the administration of SARS-CoV-2 spike S1 protein. **(A)**, Representative confocal microscopy images of lung tissue exhibiting truncated (active form) caspase 3 (green fluorescence), type I alveolar cells (AT1, red fluorescence), and type II alveolar cells (AT2, white fluorescence). **(B)**, Quantitative summary of the relative extents of stain-positive area (%) or numbers of cells in the three treatment groups. Data presented are means ± the standard error of the mean (SEM) (n = 5 per group). * *p* < 0.05 compared with the S1+saline group; one-way analysis of variance (ANOVA) followed by Student–Newman–Keuls (SNK) *t*-test. Treatment groups are as described in Figure 1.

Infection with SARS-CoV-2 results in the apoptosis of alveolar epithelial cells and contributes to lung injury (Mulay et al., 2021). As shown in Figure 2, treatment with S1 induced apoptosis of the alveolar epithelial cells throughout the infected tissue of the lung, especially in the bronchioles and alveoli made up of AT1 and AT2 alveolar cells. However, treatment with NRICM101 significantly reduced the S1-induced lung-tissue cell death and also restored the abundance of AT1 cells. Notably, the abundance of AT2 cells was greatly increased in the S1+NRICM101 group relative to both the S1+saline and sham groups. This shows that NRICM101 significantly inhibited S1-induced alveolar cell apoptosis in hACE2 mice.

### 3.3 NRICM101 reduced SARS-CoV-2 spike protein S1-induced pulmonary inflammation and leukocyte infiltration in hACE2 mice

**FIGURE 3:**
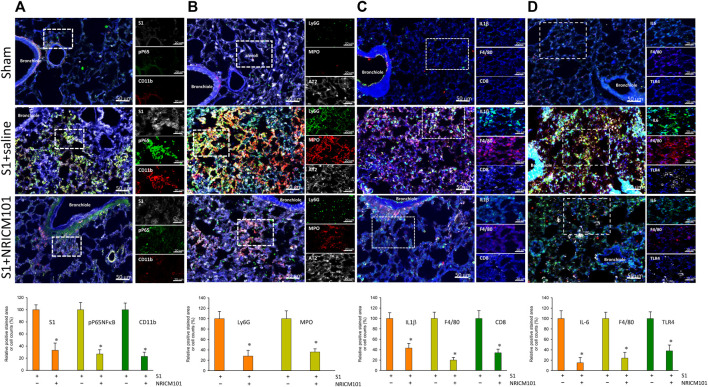
The effects of NRICM101 after 72 h on S1-induced pulmonary inflammation and leukocyte infiltration in K18-hACE2 mice with lung injury symptoms induced by the administration of SARS-CoV-2 spike S1 protein. Representative confocal microscopy images of the lung tissue revealing: **(A)**, phospho-P65NFκB (active form) (green fluorescence), CD11b leukocytes (red fluorescence), and S1 protein (S1, white fluorescence); **(B)**, Ly6G (neutrophil marker) (green fluorescence), myeloperoxidase (MPO, red fluorescence), and type II alveolar cells (AT2, white fluorescence); **(C)**, IL-1β (green fluorescence), F4/80 monocytes (red fluorescence), and CD8 lymphocytes (white fluorescence); and **(D)**, IL-6 (green fluorescence), F4/80 monocytes (red fluorescence), and TLR4 (white fluorescence). Bottom row of panels: quantitative summaries of the relative extents of stain-positive area (%) or numbers of cells in the three treatment groups. Data presented are mean ± the standard error of the mean (SEM) (n = 5 per group). * *p* < 0.05 compared with the S1+saline group; one-way analysis of variance (ANOVA) followed by Student–Newman–Keuls (SNK) *t*-test. Treatment groups are as described in Figure 1.

The dysregulation of the cytokine response that is induced by SARS-CoV-2 is known to cause abnormal inflammation leading to cell death and lung injury (Mehta et al., 2020; Rodrigues et al., 2020; Varga et al., 2020; Karki et al., 2021). We next investigated whether the protective effects of NRICM101 against SARS-CoV-2-induced lung injury operate via suppression of both inflammation and this S1-induced dysregulation of the cytokine response. Immunohistochemical examination revealed that NRICM101 significantly reduced S1 levels in the lung tissues and attenuated the S1-induced increase in leukocytes, including neutrophils and monocytes/macrophages, as evidenced by positive staining by CD11b (Figure 3A), Ly6G (Figure 3B), and F4/80 (Figures 3C, D), respectively. Moreover, the S1-induced inflammatory response was also strongly suppressed in the S1+NRICM101 group, as evidenced by elevated levels of phosphorylated activated nuclear factor-kappa B (NF-κB) pp65 (Figure 3A), myeloperoxidase (MPO; Figure 3B), IL-1β (Figure 3C), and Toll-like receptor 4 (TLR4) and IL-6 (Figure 3D), relative to the S1+saline group. These results suggest that NRICM101 can ameliorate S1-induced leukocyte infiltration and inflammatory responses in the lung tissues of K18-hACE2 mice.

### 3.4 NRICM101 inhibits SARS-CoV-2 S1-stimulated gene expression in hACE2 mice

**FIGURE 4:**
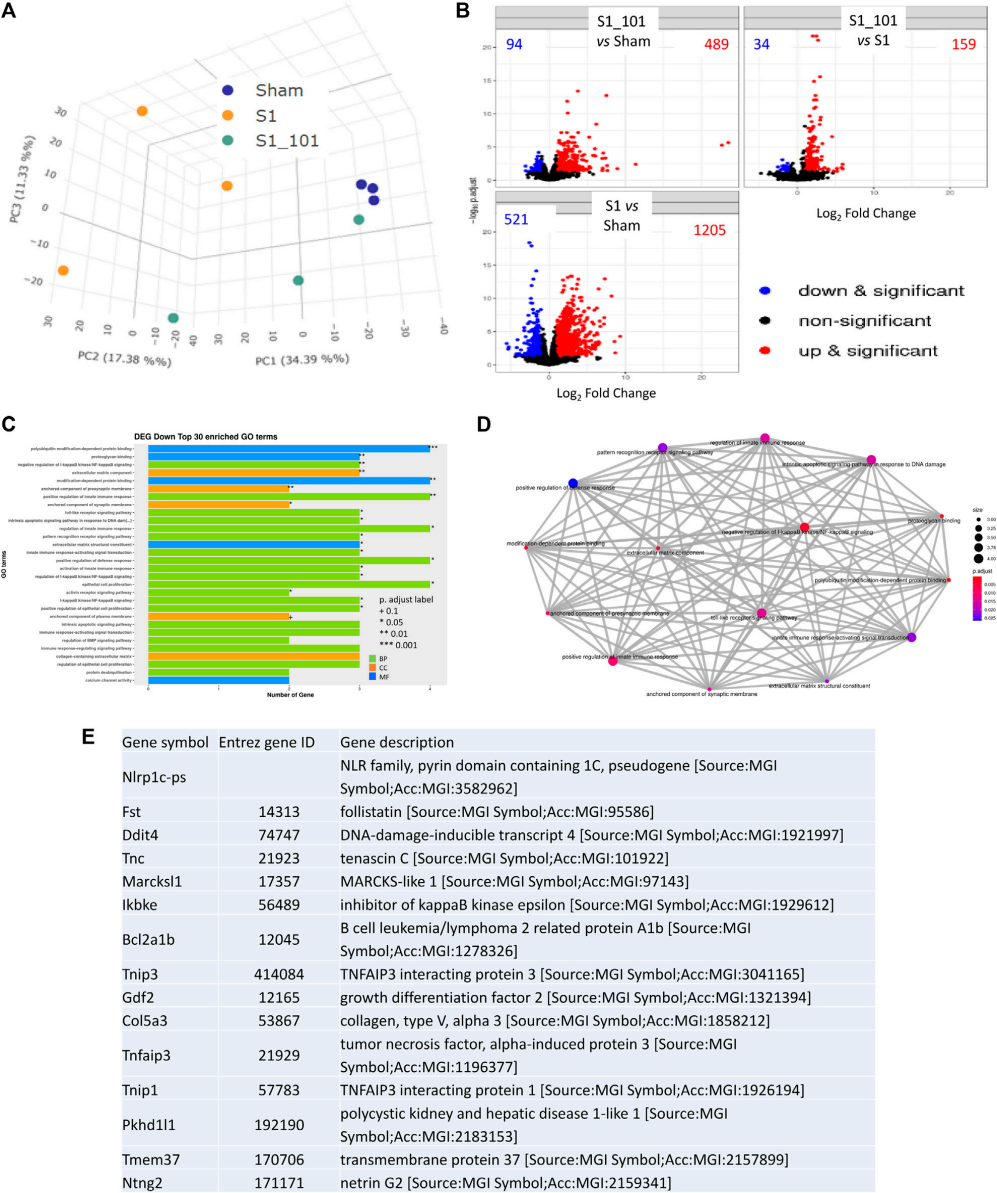
The effects of NRICM101 after 72 h on S1-induced gene expression levels in K18-hACE2 mice with lung injury symptoms induced by the administration of SARS-CoV-2 spike S1 protein. We assessed changes in gene expression using next-generation sequencing (NGS) and reverse-transcription polymerase chain reaction (RT-PCR) assays to identify differentially expressed genes (DEGs) in the S1+NRICM101 group versus the S1+saline group. **(A)**, A principal component analysis (PCA) plot of the DEG profiles of the treatment groups. **(B)**, A Volcano plot showing the DEGs between different treatment groups; comparison between S1+NRICM101 and sham (upper left), S1+NRICM101 and S1+saline (upper right), S1+saline and sham (lower left). **(C)**, The top 30 enriched gene ontology (GO) terms related to the downregulated DEGs. **(D)**, A network plot of the GO terms related to the downregulated DEGs. **(E)**, The top 15 downregulated DEGs.

To further investigate the molecular mechanisms underlying the protective effects of NRICM101, we examined differentially expressed genes (DEGs) using next-generation sequencing (NGS) and reverse-transcription polymerase chain reaction (RT-PCR) assays to identify targets that may be specifically affected by NRICM101. To do this, we compared gene expression between the S1+NRICM101 and S1+saline groups. We defined DEGs as those genes that had an adjusted *p* < 0.005. We identified 1726 genes whose expression was different in the S1+saline groups from that in the sham group. Of these, the expression levels of 1205 (69.8%) were higher and those of 521 (30.2%) were lower. We conducted a principal component analysis (PCA) to produce a visual representation of how the three treatment groups were related in terms of their gene expression patterns. The S1+NRICM101 group was more similar to the sham group than the S1+saline group (Figure 4A). We then produced volcano plots showing which DEGs were up- and downregulated in different comparisons between the groups (Figure 4B). The top 30 enriched downregulated gene ontology (GO) terms between the S1+NRICM101 and S1+saline groups are shown in Figure 4C. The top thirty downregulated GO terms had adjusted *p* as shown in the figure; these thirty were thus assumed to be significantly enriched (Figure 4C). They are strongly associated with immune-related functions, which suggests that the S1+NRICM101 group exhibited a reduced immune response. Specifically, the genes whose expression was significantly reduced in response to NRICM101 treatment are associated with the innate immune response, pattern recognition receptor (PRR), and TLR signaling pathways (Figure 4D). The TLR signaling pathway is known to be both involved in inflammation and dysregulated under COVID-19 infection; specifically, evidence has shown that the inflammation that is induced by the SARS-CoV-2 spike protein is activated via TLR2 and TLR4.

The top 15 downregulated genes in the S1+ NRICM101 group are shown in Figure 4E. Of these, DNA-damage-inducible transcript 4 (*Ddit4*) is also known as protein regulated in development and DNA damage response 1 (REDD1). *Ddit4* expression has been shown to be activated by DNA damage (Ellisen et al., 2002) and energy stress (McGhee et al., 2009). Inhibitor of nuclear factor kappa B kinase subunit epsilon (IKBKE, also known as IKKE, IKKI, and IKK-E) is a noncanonical I-kappa-B kinase (IKK) that is essential for regulating antiviral signaling pathways. It is also identified as mediating the signaling involved in the NOD-like receptor, TLR, IL-17, C-type lectin, and Rig-I-like receptor signaling pathways in the KEGG pathway database. TNF-α induced protein 3 (TNFAIP3**,** also known as A20, AISBL, AIFBL1, OTUD7C, and TNFA1P2) is identified as a gene whose expression is rapidly induced by tumor necrosis factor (TNF). This gene and its associated protein are identified as involved in the cytokine-mediated immune and inflammatory responses and necroptosis in the KEGG pathway database.

### 3.5 Inhibitory activity of NRICM101 against the ACE2 and the various spike proteins interaction

**FIGURE 5:**
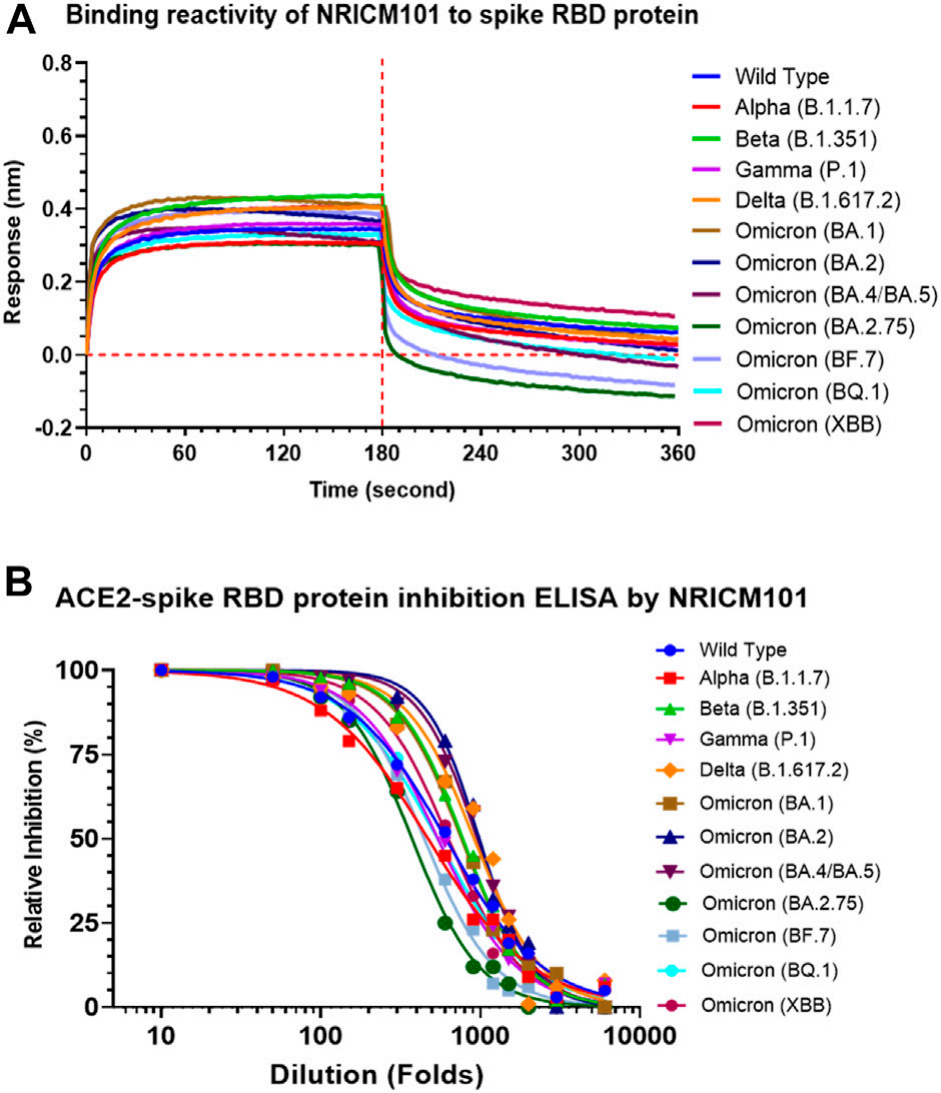
The activities of NRICM101 that act to protect against SARS-CoV-2. **(A)**, A bio-layer interferometry (BLI) assay revealing the ability of NRICM101 to bind to the spike receptor-binding domain (RBD) proteins of the following SARS-CoV-2 variants: the wild type, Alpha (B.1.1.7), Beta (B.1.351), Gamma (P.1), Delta (B.1.617.2), Omicron (B.1.1529), Omicron (BA.2), Omicron (BA.4/BA.5), Omicron (BA.2.75), Omicron (BF.7), Omicron (BQ.1), and Omicron (XBB). **(B)**, An enzyme-linked immunosorbent assay (ELISA) showing the inhibition of the binding of the spike RBDs of the same SARS-CoV-2 variants with human angiotensin-converting enzyme 2 (ACE2) by various dilutions of NRICM101. We calculated the percentage inhibition based on the binding signal normalized to that of the same spike RBD in the absence of NRICM101 treatment.

We examined the anti-SARS-CoV-2 effects of NRICM101 in terms of its binding affinity with the spike receptor-binding domain (RBD) proteins of several variants and its inhibition of the spike protein–ACE2 interaction. As shown in Figure 5A, a bio-layer interferometry (BLI) assay indicated that NRICM101 was able to bind to all the spike RBD protein variants we tested. We also conducted an enzyme-linked immunosorbent assay (ELISA), and this revealed that NRICM101 can disrupt the binding of all the RBD variants we tested to human ACE2 (Figure 5B). For the variants we tested, the half maximal effective concentration (EC_50_) of NRICM101 (diluted in PBS) was 0.165 mg/mL (wild type), 0.213 mg/mL (Alpha), 0.129 mg/mL (Beta), 0.185 mg/mL (Gamma), 0.110 mg/mL (Delta), 0.132 mg/mL (Omicron), 0.102 mg/mL (BA.2), 0.105 mg/mL (BA.4/BA.5), 0.269 mg/mL Omicron (BA.2.75), 0.225 mg/mL Omicron (BF.7), 0.186 mg/mL Omicron (BQ.1), and 0.159 mg/mL Omicron (XBB). Thus, NRICM101 exhibits strong anti-SARS-CoV-2 properties, despite the existence of many variants of this virus.

### 3.6 NRICM101 attenuated LPS-induced production of cytokines and chemokines in murine alveolar macrophages

**FIGURE 6:**
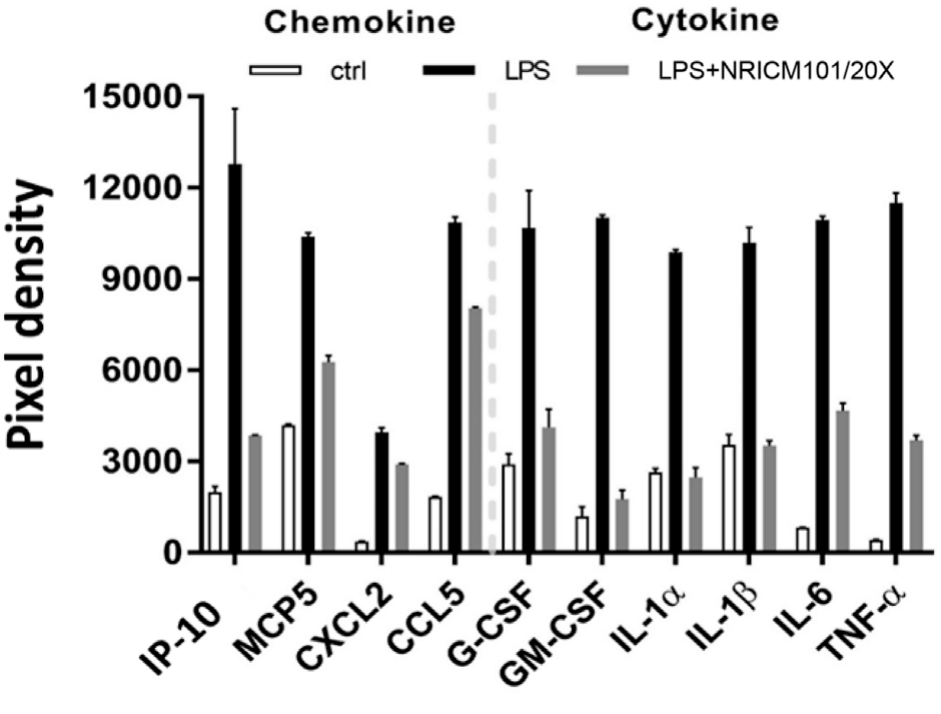
Inhibition of LPS-induced cytokine and chemokine production in murine alveolar macrophages by NRICM101. We used a 20-fold dilution of NRICM101 in this assay.

The above results showed that NRICM101 exhibits excellent anti-inflammatory activity in the lung tissues of S1-treated K18-hACE2 mice, and our previous research has shown that it can suppress the LPS-induced production of IL-6 and TNF-α in murine alveolar macrophages (Tsai et al., 2021). To further investigate its regulatory effects on LPS-induced inflammation, we analyzed the profile of inflammation-related cytokines in LPS-stimulated murine alveolar macrophages that had or had not been treated with NRICM101. As shown in Figure 6, NRICM101 significantly suppressed the expression of several LPS-induced cytokines and chemokines, including TNF-α, IL-1α, IL-1β, IL-6, IP-10, MCP-5, CXCL2, CCL5, GM-CSF, and G-CSF.

### 3.7 RNA-seq profiles of alveolar macrophages activated by LPS

**FIGURE 7:**
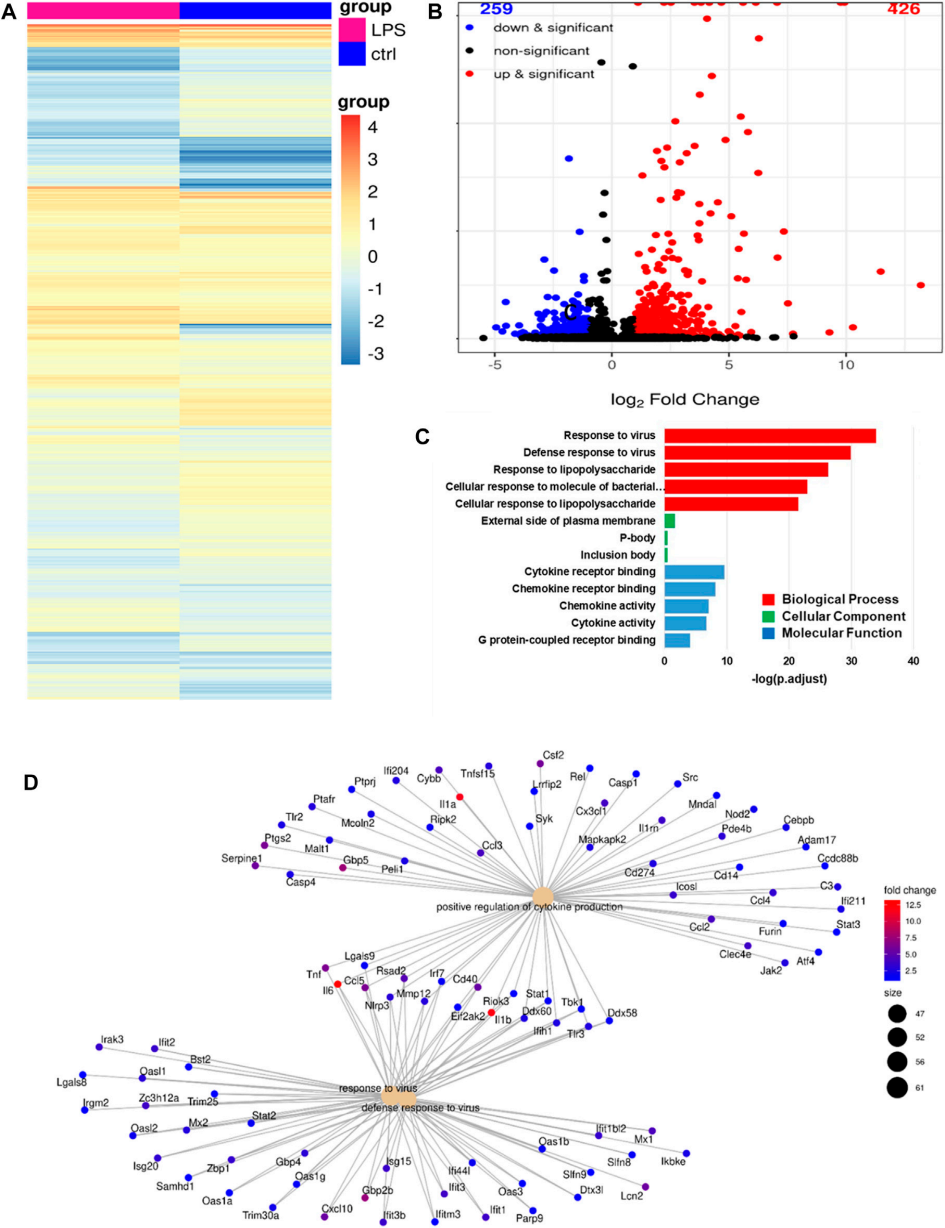
RNA-seq analysis revealing the phenotype of LPS-activated alveolar macrophages. **(A)**, A heatmap of the differentially expressed genes (DEGs) in LPS-activated versus untreated alveolar macrophages. **(B)**, A volcano plot showing the same DEGs as in (**A**). **(C)**, The top 13 gene ontology (GO) terms associated with the upregulated DEGs from (**A**). **(D)**, A cnetplot of the upregulated DEGs from (**A**).

We analyzed the RNA-seq profiles of the mRNA expressed by alveolar macrophages that were activated by LPS to identify the transcriptional changes they had undergone. The first step was to identify the DEGs in LPS-stimulated monocytes versus untreated control alveolar macrophages. As before, DEGs were defined as those for which adjusted *p* < 0.005. In total, we identified 685 DEGs, 426 (62%) of which were upregulated and 259 (38%) of which were downregulated, as shown in the gene expression heatmap in Figure 7A and in the volcano plot in Figure 7B. In Figure 7C, we show a group of 13 selected enriched GO terms that were upregulated in the LPS-stimulated cells. The top three of these GO terms were considered significant because they had adjusted *p* < 0.005. These terms included cellular response to bacterial molecules, cellular response to LPS, viral response, antiviral response, and LPS response. Thus, these terms were strongly linked to the immune response, revealing that the immune response of the LPS-treated cells had increased. We performed a category netplot (cnetplot) analysis to visualize the linkages between the genes and biological concepts (Figure 7D). This showed that the genes that were upregulated in response to LPS stimulation were involved in the signaling pathways of the antiviral defense response, positive regulation of cytokine production, and the response to viruses (Figure 7D).

### 3.8 Effects of LPS stimulation on alveolar macrophages were inhibited by NRICM101

**FIGURE 8:**
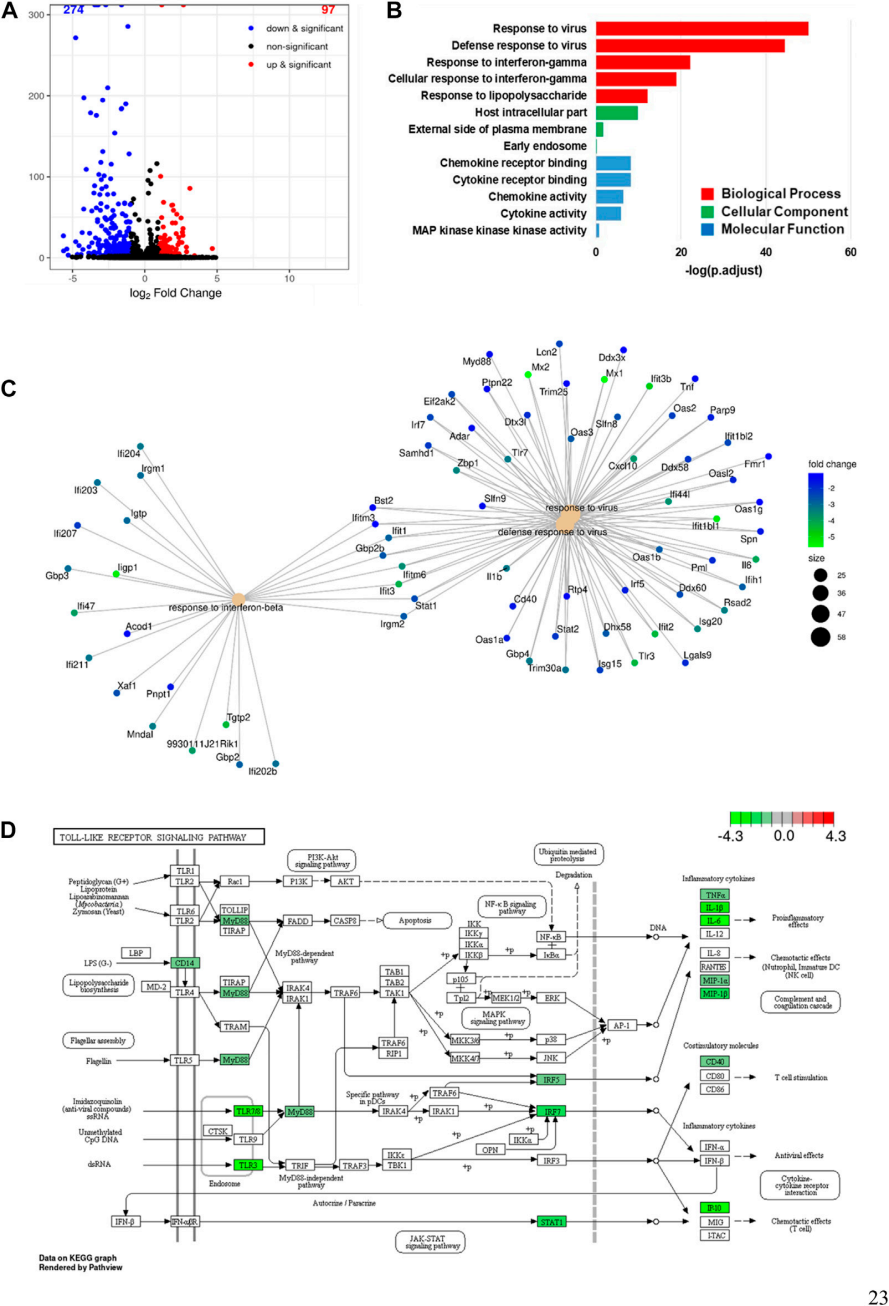
Inhibitory activities of NRICM101 toward LPS-stimulated alveolar macrophages. (**A**), A volcano plot of the differentially expressed genes (DEGs) in alveolar macrophages treated with LPS versus those treated with LPS and NRICM101. **(B)**, The top 13 gene ontology (GO) terms associated with the downregulated DEGs from (**A**). **(C)**, A cnetplot of the downregulated DEGs from (**A**). **(D)**, KEGG pathway analysis showing how NRICM101 inhibits the Toll-like receptor (TLR) signaling pathway. Genes in the green boxes are significantly downregulated by treatment with NRICM101.

We identified DEGs in alveolar macrophages treated with LPS+NRICM101 and LPS only, once again defining genes that had adjusted *p* < 0.005 as DEGs. In total, we found 371 DEGs, 274 of which (74%) were downregulated and 97 of which (26%) were upregulated in the cells treated with LPS+NRICM101 relative to those treated with LPS only, as shown in the volcano plot in Figure 8A. The 13 enriched GO terms shown in Figure 8B were downregulated, and the top three had adjusted *p* < 0.005. These terms include IFN-γ response, cellular IFN-γ response, viral response, antiviral response, and LPS response (Figure 8B). All of these terms are thus strongly associated with the immune response, implying that the cells in the LPS+NRICM101 treatment exhibited a diminished immune response. The genes whose expression decreased in response to treatment with NRICM101 are associated with pathways linked to antiviral response, INF-β response, and viral response (Figure 8C). According to a previous study, the inflammatory TLR signaling pathway becomes dysregulated under COVID-19 infection (Tseng et al., 2022). We conducted a KEGG pathway analysis of the DEGs we had identified in this final analysis to investigate the role of NRICM101 in the TLR signaling pathway (Figure 8D). As the KEGG analysis shows, the NRICM101 suppressed the production of inflammatory cytokines that is stimulated by LPS. These cytokines include IL-1β, IL-6, TNF-α, MIP-1β, IP-10, MIP-1α. It also suppressed the costimulatory molecule CD40 via the regulation of several other pathways: MyD88-dependent, MyD88-independent, PI3K–Akt, and JAK–STAT.

## 4 Discussion

Current clinical and laboratory evidence indicates that SARS-CoV-2-induced lung pathology can be attributed to the combined effects of the direct cytopathic activity of the virus and the dysregulated inflammatory and immune responses that are indirect effects of the virus. Autopsy examinations of patients who have died from COVID-19 reveal that DAD is the most common type of lung injury suffered by these patients (Lamers and Haagmans, 2022). This DAD exhibits signs of the exudative phase, such as edema (both interstitial and intra-alveolar), abnormal pneumocyte apoptosis, hyaline membranes, congestion of the capillaries, and microvascular thrombosis (Carsana et al., 2020; Menter et al., 2020). The abnormal pneumocyte apoptosis has been confirmed by immunostaining the diseased lungs, which contained fewer AT2 and AT1 cells than healthy lungs (Chen et al., 2020; Delorey et al., 2021; Melms et al., 2021).

In this study, using the SARS-CoV-2 spike protein S1 to induce pulmonary injury in hACE2 mice, we successfully established an animal model displaying COVID-19-associated pneumonia (pulmonary injury) and ARDS. We observed severe pulmonary injury in the S1-challenged mice, with a dramatic drop in their SO_2_. This pulmonary injury exhibited several of the features of DAD, including strong exudation, edema (both interstitial and intra-alveolar), hyaline membranes, abnormal pneumocyte apoptosis (AT1 and AT2), capillary congestion, and strong inflammation (leukocyte infiltration and cytokine production).

All these DAD features improved significantly as a result of post-treatment with NRICM101 at a dose of 3.0 g/kg (orally administered), which is analogous to the usual clinical dose. Our histological observations showed that in the S1+NRICM101 group, the alveolar damage and collapse of spaces had been effectively prevented: the alveoli were intact and the fragile alveolar septa still included their narrow capillaries, which were lined with flattened AT1 cells and cuboidal AT2 cells. The AT2 cells had retained the potential to self-renew and differentiate into AT1 cells. These features were easily observable throughout the lung tissue and the SO_2_ was restored to almost the same level as in the sham group. We also observed that the abundance of AT2 cells was significantly greater in the S1+NRICM101 group than in the S1+saline group, suggesting that NRICM101 may play a role in activating AT2 activity, thus facilitating lung repair.

Accompanying these pathological data, our RNA-seq data analysis revealed that the DAD features in the S1+saline group were strongly associated with signaling pathways linked to activating the innate immune response, PRR, positive regulation of the defense response, and TLR. All of these pathways were downregulated by treatment with NRICM101. Combined, these data suggest that using NRICM101 to treat COVID-19-related pulmonary injury is a reasonable and successful approach.

We also examined the activity of NRICM101 against LPS-induced inflammation. Our results showed that the regulatory effects of NRICM101 against the dysregulation of cytokines and inflammation can be observed not only in S1-treated lung tissue but also in LPS-treated murine alveolar macrophages. The cytokine storm or dysregulated cytokine immune response is not unique to patients with SARS-CoV-2 (Fajgenbaum and June, 2020), but it can be life threatening. Our results indicate that NRICM101 may be also have potential for treating immune-dysregulation–related diseases.

We acknowledge that S1 is insufficient to fully represent all the pathologies of SARS-CoV-2, the spike protein and its S1 subunit have been demonstrated to play a key role in viral pathogenesis, evolution, and transmission. However, some vaccines may instead use highly immunogenic regions like the RBD (receptor binding domain) found within the S1 subunit (Martínez-Flores et al., 2021), as the RBM (receptor binding motif) within the RBD directly interacts with the ACE2 receptor (Shang et al., 2020). The RBD in S1 has received considerable attention since it serves as an intermediary factor in the virus-host cell interaction, specifically by binding to the ACE2 receptor of the host cell. This binding is critical to the viral infection process, and as a result, most of the mutations of SARS-CoV-2 occur within the RBD of S1. Besides, the spike protein S1 subunit is the key immune stimulant in the human body’s response to SARS-CoV-2, many strategies, such as antibodies against COVID-19 disorder, have been designed according to anti-S1/RBD. Therefore, in this study, we used the S1 protein - which is the key protein subunit responsible for invading the human system through the ACE2 receptor with the same protein sequence as the original Wuhan strain - as a stimulator to mimic the way SARS-CoV-2 behaves. There are many advantages to using this approach, including the ease and safety of experimental handling, the quick development of a disease model for COVID-19, and the ability to observe typical pathological changes in histology (such as diffused alveolar damage or DAD) and immunology (such as cytokine storm, NET formation, TLR activation, and more). We suggest that the S1-induced animal model represents some but not all of the pathologies of SARS-CoV-2 and is suitable for the quick development and analysis of new drugs against SARS-CoV-2.

SARS-CoV-2-associated respiratory illness is the main challenge for patients with COVID-19, and it can develop into life-threatening pneumonia. However, there is still no effective pharmacologic treatment for SARS-CoV-2-associated ARDS. Our previous study revealed the clinical effectiveness of NRICM101 for treating COVID-19 patients (Tsai et al., 2021). In the present study, we have demonstrated that it can effectively suppress lung inflammation and ameliorate lung injury in SARS-CoV-2 spike protein S1-treated K18-hACE2 mice. These findings provide some mechanistic insights into how NRICM101 functions when used to treat COVID-19.

## Data Availability

The original contributions presented in the study are publicly available. This data can be found here: https://www.ncbi.nlm.nih.gov/PRJNA975698.

## References

[B1] Amirian E. S. (2020). Potential fecal transmission of SARS-COV-2: Current evidence and implications for public health. Int. J. Infect. Dis. 95, 363–370. 10.1016/j.ijid.2020.04.057 32335340 PMC7195510

[B2] Beyerstedt S. Casaro E. B. Rangel É. B. (2021). Covid-19: Angiotensin-converting enzyme 2 (ACE2) expression and tissue susceptibility to SARS-COV-2 infection. Eur. J. Clin. Microbiol. Infect. Dis. 40 (5), 905–919. 10.1007/s10096-020-04138-6 33389262 PMC7778857

[B3] Carsana L. Sonzogni A. Nasr A. Rossi R. S. Pellegrinelli A. Zerbi P. (2020). Pulmonary post-mortem findings in a series of COVID-19 cases from northern Italy: A two-centre descriptive study. Lancet Infect. Dis. 20 (10), 1135–1140. 10.1016/s1473-3099(20)30434-5 32526193 PMC7279758

[B4] Chen J. Wu H. Yu Y. Tang N. (2020). Pulmonary alveolar regeneration in adult COVID-19 patients. Cell. Res. 30 (8), 708–710. 10.1038/s41422-020-0369-7 32632255 PMC7338112

[B5] Colunga Biancatelli R. M. Solopov P. A. Sharlow E. R. Lazo J. S. Marik P. E. Catravas J. D. (2021). The SARS-CoV-2 spike protein subunit S1 induces COVID-19-like acute lung injury in Κ18-hACE2 transgenic mice and barrier dysfunction in human endothelial cells. Am. J. Physiol. Lung Cell. Mol. Physiol. 321 (2), L477–L484. 10.1152/ajplung.00223.2021 34156871 PMC8384477

[B6] Delorey T. M. Ziegler C. G. Heimberg G. Normand R. Yang Y. Segerstolpe Å. (2021). COVID-19 tissue atlases reveal SARS-CoV-2 pathology and cellular targets. Nature 595 (7865), 107–113. 10.1038/s41586-021-03570-8 33915569 PMC8919505

[B7] Ellisen L. W. Ramsayer K. D. Johannessen C. M. Yang A. Beppu H. Minda K. (2002). REDD1, a developmentally regulated transcriptional target of p63 and p53, links p63 to regulation of reactive oxygen species. Mol. Cell. 10 (5), 995–1005. 10.1016/s1097-2765(02)00706-2 12453409

[B8] Fajgenbaum D. C. June C. H. (2020). Cytokine storm. N. Engl. J. Med. 383 (23), 2255–2273. 10.1056/nejmra2026131 33264547 PMC7727315

[B9] Gheblawi M. Wang K. Viveiros A. Nguyen Q. Zhong J. C. Turner A. J. (2020). Angiotensin-converting enzyme 2: SARS-CoV-2 receptor and regulator of the renin-angiotensin system: Celebrating the 20th anniversary of the discovery of ACE2. Circ. Res. 126 (10), 1456–1474. 10.1161/circresaha.120.317015 32264791 PMC7188049

[B10] Grasselli G. Tonetti T. Protti A. Langer T. Girardis M. Bellani G. (2020). Pathophysiology of COVID-19-associated acute respiratory distress syndrome: A multicentre prospective observational study. Lancet Respir. Med. 8 (12), 1201–1208. 10.1016/s2213-2600(20)30370-2 32861276 PMC7834127

[B11] Guan W. J. Ni Z. Y. Hu Y. Liang W. H. Ou C. Q. He J. X. (2020). Clinical characteristics of coronavirus disease 2019 in China. N. Engl. J. Med. 382 (18), 1708–1720. 10.1056/nejmoa2002032 32109013 PMC7092819

[B12] Harvey W. T. Carabelli A. M. Jackson B. Gupta R. K. Thomson E. C. Harrison E. M. (2021). SARS-CoV-2 variants, Spike mutations and immune escape. Nat. Rev. Microbiol. 19 (7), 409–424. 10.1038/s41579-021-00573-0 34075212 PMC8167834

[B13] Hsieh P. C. Chao Y. C. Tsai K. W. Li C. H. Tzeng I. S. Wu Y. K. (2022). Efficacy and safety of complementary therapy with jing Si herbal tea in patients with mild-to-moderate COVID-19: A prospective cohort study. Front. Nutr. 9, 832321. 10.3389/fnut.2022.832321 35369061 PMC8967163

[B14] Huang C. Wang Y. Li X. Ren L. Zhao J. Hu Y. (2020). Clinical features of patients infected with 2019 novel coronavirus in Wuhan, China. Lancet 395 (10223), 497–506. 10.1016/s0140-6736(20)30183-5 31986264 PMC7159299

[B15] Indari O. Jakhmola S. Manivannan E. Jha H. C. (2021). An update on antiviral therapy against SARS-CoV-2: How far have we come? Front. Pharmacol. 12, 632677. 10.3389/fphar.2021.632677 33762954 PMC7982669

[B16] Karki R. Sharma B. R. Tuladhar S. Williams E. P. Zalduondo L. Samir P. (2021). Synergism of TNF-α and IFN-γ triggers inflammatory cell death, tissue damage, and mortality in SARS-CoV-2 infection and cytokine shock syndromes. Cell. 184 (1), 149–168.e17. 10.1016/j.cell.2020.11.025 33278357 PMC7674074

[B17] Lamers M. M. Haagmans B. L. (2022). SARS-CoV-2 pathogenesis. Nat. Rev. Microbiol. 20 (5), 270–284. 10.1038/s41579-022-00713-0 35354968

[B18] Li W. Moore M. J. Vasilieva N. Sui J. Wong S. K. Berne M. A. (2003). Angiotensin-converting enzyme 2 is a functional receptor for the SARS coronavirus. Nature 426 (6965), 450–454. 10.1038/nature02145 14647384 PMC7095016

[B19] Lu R. Zhao X. Li J. Niu P. Yang B. Wu H. (2020). Genomic characterisation and epidemiology of 2019 novel coronavirus: Implications for virus origins and receptor binding. Lancet 395 (10224), 565–574. 10.1016/s0140-6736(20)30251-8 32007145 PMC7159086

[B20] Martínez-Flores D. Zepeda-Cervantes J. Cruz-Reséndiz A. Aguirre-Sampieri S. Sampieri A. Vaca L. (2021). SARS-CoV-2 vaccines based on the spike glycoprotein and implications of new viral variants. Front. Immunol. 12, 701501. 10.3389/fimmu.2021.701501 34322129 PMC8311925

[B21] McGhee N. K. Jefferson L. S. Kimball S. R. (2009). Elevated corticosterone associated with food deprivation upregulates expression in rat skeletal muscle of the mTORC1 repressor, REDD1. J. Nutr. 139 (5), 828–834. 10.3945/jn.108.099846 19297425 PMC2714387

[B22] Mehta P. McAuley D. F. Brown M. Sanchez E. Tattersall R. S. Manson J. J. (2020). COVID-19: Consider cytokine storm syndromes and immunosuppression. Lancet 395 (10229), 1033–1034. 10.1016/s0140-6736(20)30628-0 32192578 PMC7270045

[B23] Melms J. C. Biermann J. Huang H. Wang Y. Nair A. Tagore S. (2021). A molecular single-cell lung atlas of lethal COVID-19. Nature 595 (7865), 114–119. 10.1038/s41586-021-03569-1 33915568 PMC8814825

[B24] Menter T. Haslbauer J. D. Nienhold R. Savic S. Hopfer H. Deigendesch N. (2020). Postmortem examination of COVID‐19 patients reveals diffuse alveolar damage with severe capillary congestion and variegated findings in lungs and other organs suggesting vascular dysfunction. Histopathology 77 (2), 198–209. 10.1111/his.14134 32364264 PMC7496150

[B25] Montazersaheb S. Hosseiniyan Khatibi S. M. Hejazi M. S. Tarhriz V. Farjami A. Ghasemian Sorbeni F. (2022). COVID-19 infection: An overview on cytokine storm and related interventions. Virol. J. 19 (1), 92. 10.1186/s12985-022-01814-1 35619180 PMC9134144

[B26] Mulay A. Konda B. Garcia G. Yao C. Beil S. Villalba J. M. (2021). SARS-CoV-2 infection of primary human lung epithelium for COVID-19 modeling and drug discovery. Cell. Rep. 35 (5), 109055. 10.1016/j.celrep.2021.109055 33905739 PMC8043574

[B27] Ping Y. H. Yeh H. Chu L. W. Lin Z. H. Hsu Y. C. Lin L. C. (2022). The traditional Chinese medicine formula Jing Guan Fang for preventing SARS-CoV-2 infection: From clinical observation to basic research. Front. Pharmacol. 13, 744439. 10.3389/fphar.2022.744439 35387343 PMC8978714

[B28] Ragab D. Salah Eldin H. Taeimah M. Khattab R. Salem R. (2020). The COVID-19 cytokine storm; what we know so far. Front. Immunol. 11, 1446. 10.3389/fimmu.2020.01446 32612617 PMC7308649

[B29] Reddy K. Hardin C. C. McAuley D. F. (2021). COVID-19–related acute respiratory distress syndrome subphenotypes and differential response to corticosteroids: Time for more precision? Am. J. Respir. Crit. Care Med. 204 (11), 1241–1243. 10.1164/rccm.202109-2213ed 34705609 PMC8786072

[B30] Ren X. Zhou J. Guo J. Hao C. Zheng M. Zhang R. (2022). Reinfection in patients with COVID-19: A systematic review. Glob. Health Res. Policy 7 (1), 12. 10.1186/s41256-022-00245-3 35488305 PMC9051013

[B31] Rendeiro A. F. Ravichandran H. Bram Y. Chandar V. Kim J. Meydan C. (2021). The spatial landscape of lung pathology during COVID-19 progression. Nature 593 (7860), 564–569. 10.1038/s41586-021-03475-6 33780969 PMC8204801

[B32] Rodrigues T. S. de Sá K. S. G. Ishimoto A. Y. Becerra A. Oliveira S. Almeida L. (2020). Inflammasomes are activated in response to SARS-CoV-2 infection and are associated with COVID-19 severity in patients. J. Exp. Med. 218 (3), 20201707. 10.1084/jem.20201707 PMC7684031 33231615

[B33] Shang J. Ye G. Shi K. Wan Y. Luo C. Aihara H. (2020). Structural basis of receptor recognition by SARS-CoV-2. Nature 581 (7807), 221–224. 10.1038/s41586-020-2179-y 32225175 PMC7328981

[B34] Sinha P. Calfee C. S. Cherian S. Brealey D. Cutler S. King C. (2020). Prevalence of phenotypes of acute respiratory distress syndrome in critically ill patients with COVID-19: A prospective observational study. Lancet Respir. Med. 8 (12), 1209–1218. 10.1016/s2213-2600(20)30366-0 32861275 PMC7718296

[B35] Smail S. W. Saeed M. Alkasalias T. Khudhur Z. O. Younus D. A. Rajab M. F. (2021). Inflammation, immunity and potential target therapy of SARS-CoV-2: A total scale analysis review. Food Chem. Toxicol. 150, 112087. 10.1016/j.fct.2021.112087 33640537 PMC7905385

[B36] Tay M. Z. Poh C. M. Rénia L. MacAry P. A. Ng L. F. (2020). The trinity of COVID-19: Immunity, inflammation and intervention. Nat. Rev. Immunol. 20 (6), 363–374. 10.1038/s41577-020-0311-8 32346093 PMC7187672

[B37] Tsai K. C. Huang Y. C. Liaw C. C. Tsai C. I. Chiou C. T. Lin C. J. (2021). A traditional Chinese medicine formula NRICM101 to target COVID-19 through multiple pathways: A bedside-to-bench study. Biomed. Pharmacother. 133, 111037. 10.1016/j.biopha.2020.111037 33249281 PMC7676327

[B38] Tseng Y. H. Lin S. J. S. Hou S. M. Wang C. H. Cheng S. P. Tseng K. Y. (2022). Curbing COVID-19 progression and mortality with traditional Chinese medicine among hospitalized patients with COVID-19: A propensity score-matched analysis. Pharmacol. Res. 184, 106412. 10.1016/j.phrs.2022.106412 36007774 PMC9395232

[B39] Varga Z. Flammer A. J. Steiger P. Haberecker M. Andermatt R. Zinkernagel A. S. (2020). Endothelial cell infection and endotheliitis in COVID-19. Lancet 395 (10234), 1417–1418. 10.1016/s0140-6736(20)30937-5 32325026 PMC7172722

[B40] Wang D. Hu B. Hu C. Zhu F. Liu X. Zhang J. (2020). Clinical characteristics of 138 hospitalized patients with 2019 novel coronavirus–infected pneumonia in Wuhan, China. JAMA 323 (11), 1061–1069. 10.1001/jama.2020.1585 32031570 PMC7042881

[B41] Wei W. C. Liaw C. C. Tsai K. C. Chiou C. T. Tseng Y. H. Chiou W. F. (2022). Targeting spike protein-induced TLR/net axis by COVID-19 therapeutic NRICM102 ameliorates pulmonary embolism and fibrosis. Pharmacol. Res. 184, 106424. 10.1016/j.phrs.2022.106424 36064077 PMC9443660

[B42] Xu Z. Shi L. Wang Y. Zhang J. Huang L. Zhang C. (2020). Pathological findings of COVID-19 associated with acute respiratory distress syndrome. Lancet Respir. Med. 8 (4), 420–422. 10.1016/s2213-2600(20)30076-x 32085846 PMC7164771

[B43] Zhang J. Wu H. Yao X. H. Zhang D. Zhou Y. Fu B. (2021). Pyroptotic macrophages stimulate the SARS-CoV-2-associated cytokine storm. Cell. Mol. Immunol. 18 (5), 1305–1307. 10.1038/s41423-021-00665-0 33742186 PMC7976727

[B44] Zhou P. Yang X. L. Wang X. G. Hu B. Zhang L. Zhang W. (2020). Addendum: A pneumonia outbreak associated with a new coronavirus of probable bat origin. Nature 588 (7836), E6. 10.1038/s41586-020-2951-z 33199918 PMC9744119

[B45] Zhu N. Zhang D. Wang W. Li X. Yang B. Song J. (2020). A novel coronavirus from patients with pneumonia in China, 2019. N. Engl. J. Med. 382 (8), 727–733. 10.1056/nejmoa2001017 31978945 PMC7092803

